# Phylogeny of *Mycoplasma bovis* isolates from Hungary based on multi locus sequence typing and multiple-locus variable-number tandem repeat analysis

**DOI:** 10.1186/1746-6148-10-108

**Published:** 2014-05-07

**Authors:** Kinga M Sulyok, Zsuzsa Kreizinger, Lilla Fekete, Szilárd Jánosi, Nóra Schweitzer, Ibolya Turcsányi, László Makrai, Károly Erdélyi, Miklós Gyuranecz

**Affiliations:** 1 Institute for Veterinary Medical Research, Centre for Agricultural Research, Hungarian Academy of Sciences, Hungária körút 21, Budapest 1143, Hungary; 2 Veterinary Diagnostic Directorate, National Food Chain Safety Office, Tábornok utca 2, Budapest 1143, Hungary; 3 Faculty of Veterinary Science, Szent István University, Hungária körút 23-25, Budapest 1143, Hungary

**Keywords:** Cattle, Genotyping, MLST, MLVA, *Mycoplasma bovis*, Phylogeny, VNTR

## Abstract

**Background:**

*Mycoplasma bovis* is an important pathogen causing pneumonia, mastitis and arthritis in cattle worldwide. As this agent is primarily transmitted by direct contact and spread through animal movements, efficient genotyping systems are essential for the monitoring of the disease and for epidemiological investigations. The aim of this study was to compare and evaluate the multi locus sequence typing (MLST) and the multiple-locus variable-number tandem repeat (VNTR) analysis (MLVA) through the genetic characterization of *M. bovis* isolates from Hungary.

**Results:**

Thirty one Hungarian *M. bovis* isolates grouped into two clades by MLST. Two strains had the same sequence type (ST) as reference strain PG45, while the other twenty nine Hungarian isolates formed a novel clade comprising five subclades. Isolates originating from the same herds had the same STs except for one case. The same isolates formed two main clades and several subclades and branches by MLVA. One clade contained the reference strain PG45 and three isolates, while the other main clade comprised the rest of the strains. Within-herd strain divergence was also detected by MLVA. Little congruence was found between the results of the two typing systems.

**Conclusions:**

MLST is generally considered an intermediate scale typing method and it was found to be discriminatory among the Hungarian *M. bovis* isolates. MLVA proved to be an appropriate fine scale typing tool for *M. bovis* as this method was able to distinguish closely related strains isolated from the same farm*.* We recommend the combined use of the two methods for the genotyping of *M. bovis* isolates. Strains have to be characterized first by MLST followed by the fine scale typing of identical STs with MLVA.

## Background

*Mycoplasma bovis* is a worldwide pathogen of intensively farmed cattle which had recently emerged as a significant infectious agent in North America and Europe [1,2]. *M. bovis* is an important causative agent of respiratory disease, mastitis and arthritis in cattle, and it was occasionally associated with genital infections and abortions as well [2-4]. This pathogen is estimated to be responsible for up to one third of the economic losses in the cattle industry caused by respiratory diseases [5].

In order to better understand the epidemiology of *M. bovis*, several typing techniques, such as random amplified polymorphic DNA (RAPD) or pulsed-field gel electrophoresis (PFGE) analysis [6-9], have been used to compare isolates. However, these techniques have their disadvantages, such as the poor reproducibility of results with the RAPD method or the need for special equipment and the time consuming nature of PFGE analysis. Recently, multi locus sequence typing (MLST) and multiple-locus variable-number tandem repeat (VNTR) analysis (MLVA) methods have been developed for the genetic characterization of *M. bovis* isolates [9,10]. MLST schemes were originally designed to capture the intermediate-level evolutionary relationships between bacterial isolates (e.g. on a country or continent level) while MLVA was proposed to detect the quick mutation events (e.g. during epidemics) within bacterial species [11,12]. To date, relatively little information is available about the application of these recently introduced assays. MLVA have been used in three *M. bovis* studies during the last years [9,13,14], while MLST was successfully applied only in one pilot study [10]. Moreover this latter typing scheme also needs to be tested with a higher number of *M. bovis* isolates.

The aim of the present study was to genetically characterize the Hungarian *M. bovis* population with the MLST and MLVA method in order to evaluate and compare these two typing systems and to better understand the epidemiology of *M. bovis* in Hungary.

## Methods

Nasal swabs, lung samples and a single lymph node were collected through routine diagnostic examinations or necropsies from different parts of Hungary between 2010 and 2013. Ethical approval was not required for the study as all samples were collected during routine diagnostic examinations or necropsies. Swabs and small pieces from the affected parts of lungs and the lymph node were homogenized in 2 ml of *Mycoplasma* broth medium (Thermo Fisher Scientific Inc./Oxoid Inc./, Waltham, MA) and cultured at 37°C. On the second day of incubation, when the slight colour change of the broth media occurred, the cultures were inoculated onto solid *Mycoplasma* media (Thermo Fisher Scientific Inc./Oxoid Inc./) and were incubated at 37°C and 5% CO_2_ for 3 days, until visible colonies appeared. *Mycoplasma* strains were filter-cloned only once before DNA extraction from 200 μl of broth culture using the DNeasy blood and tissue kit (Qiagen Inc., Hilden, Germany). In order to identify the isolates, PCR targeting the *uvrC* gene of *M. bovis* was performed on all samples [15]. The thirty-one strains isolated and analyzed in this study are listed in Table 1.

**Table 1:** **Background data for the thirty-one *M. bovis *** **strains analyzed in this study**

**Sample ID**	**Herd of origin**	**Date**	**Sample type**	**GenBank accession numbers**				**Copy numbers of tandem repeats**								
				***fusA***	***gyrB***	***lepA***	***rpoB***	**TR14**	**TR29**	**TR30**	**TR31**	**TR35**	**TR40-41**	**TR49-51**	**TR52**	**TR59**
PG45	Connecticut	1961	Lung	JQ031392	JQ031411	JQ031435	JQ031493	2.2	4.8	2.3	5.8	4.0	4.0	2.2	4.0	2.4
MYC2	Püspökhatvan	2011	Lung	KF926436	KF926437	KF926438	KF926439	-	-	3.3	-	3.0	-	1.2	-	-
MYC22	Sümeg	2012	Lung	KF926440	KF926441	KF926442	KF926443	-	0.8	3.3	-	3.0	8.0	1.2	3.0	2.4
MYC30	Bugyi	2012	Lung	KJ438237	KJ438174	KJ438195	KJ438216	-	0.8	2.3	5.8	3.0	8.0	1.2	3.0	2.4
MYC42	Nemti	2012	Lung	KJ438238	KJ438175	KJ438196	KJ438217	-	12.8	3.3	5.8	3.0	8.0	1.2	4.0	2.4
MYC43	Zsana	2012	Lung	KF926444	KF926445	KF926446	KF926447	-	2.8	3.3	-	3.0	11.0	1.2	3.0	2.4
MYC44	Győrszentiván	2012	Lung	KJ438239	KJ438176	KJ438197	KJ438218	-	2.8	3.3	-	3.0	-	0.6	3.0	1.4
MYC45	Budapest	2012	Lung	KJ438240	KJ438177	KJ438198	KJ438219	-	2.8	3.3	-	3.0	11.0	0.6	3.0	1.4
MYC46	Budapest	2012	Lung	KF926448	KF926449	KF926450	KF926451	-	2.8	3.3	-	3.0	11.0	0.6	3.0	1.4
MYC47	Dabas	2012	Lung	KJ438241	KJ438178	KJ438199	KJ438220	-	2.8	3.3	-	3.0	11.0	1.2	3.0	-
MYC48	Ősi	2012	Nasal swab	KJ438242	KJ438179	KJ438200	KJ438221	-	-	3.3	-	3.0	-	1.2	-	-
MYC49	Ősi	2012	Nasal swab	KF926452	KF926453	KF926454	KF926455	-	12.8	3.3	-	3.0	8.0	1.2	4.0	2.4
MYC50	Ősi	2012	Lung	KJ438243	KJ438180	KJ438201	KJ438222	-	12.8	3.3	-	3.0	8.0	1.2	4.0	-
MYC51	Ősi	2012	Nasal swab	KJ438244	KJ438181	KJ438202	KJ438223	-	12.8	3.3	-	3.0	8.0	1.2	4.0	2.4
MYC52	Solt	2012	Lung	KJ438245	KJ438182	KJ438203	KJ438224	2.2	4.8	2.3	7.8	4.0	4.0	2.2	4.0	2.4
MYC53	Solt	2012	Lung	KF926456	KF926457	KF926458	KF926459	2.2	4.8	2.3	7.8	4.0	4.0	2.2	4.0	2.4
MYC65	Csengersima	2012	Nasal swab	KJ438246	KJ438183	KJ438204	KJ438225	-	1.8	3.3	5.8	3.0	8.0	1.2	4.0	2.4
MYC66	Csengersima	2012	Nasal swab	KJ438247	KJ438184	KJ438205	KJ438226	-	-	3.3	5.8	3.0	8.0	1.2	6.0	2.4
MYC67	Csengersima	2012	Lung	KF926460	KF926461	KF926462	KF926463	-	-	3.3	5.8	3.0	8.0	1.2	6.0	2.4
MYC68	Csengersima	2012	Lung	KJ438248	KJ438185	KJ438206	KJ438227	-	-	3.3	5.8	3.0	8.0	1.2	6.0	2.4
MYC69	Komárom	2013	Nasal swab	KJ438249	KJ438186	KJ438207	KJ438228	-	1.8	3.3	-	3.0	8.0	1.2	4.0	-
MYC70	Komárom	2013	Nasal swab	KJ438250	KJ438187	KJ438208	KJ438229	-	1.8	3.3	4.8	3.0	8.0	1.2	4.0	-
MYC71	Komárom	2013	Nasal swab	KJ438251	KJ438188	KJ438209	KJ438230	-	1.8	3.3	-	3.0	8.0	1.2	4.0	-
MYC72	Komárom	2013	Nasal swab	KJ438252	KJ438189	KJ438210	KJ438231	-	1.8	3.3	4.8	3.0	8.0	1.2	4.0	-
MYC73	Komárom	2013	Nasal swab	KJ438253	KJ438190	KJ438211	KJ438232	-	1.8	3.3	4.8	3.0	8.0	1.2	4.0	-
MYC74	Komárom	2013	Nasal swab	KF926464	KF926465	KF926466	KF926467	-	1.8	3.3	4.8	-	8.0	1.2	4.0	-
MYC75	Komárom	2013	Nasal swab	KJ438254	KJ438191	KJ438212	KJ438233	-	1.8	3.3	-	3.0	8.0	1.2	4.0	-
MYC76	Komárom	2013	Nasal swab	KJ438255	KJ438192	KJ438213	KJ438234	-	1.8	3.3	4.8	-	8.0	1.2	4.0	-
MYC77	Kertészsziget	2010	Lung	KJ438256	KJ438193	KJ438214	KJ438235	2.2	0.8	3.3	-	3.0	11.0	0.6	3.0	2.4
MYC78	Hosszúpályi	2011	Lung	KF926468	KF926469	KF926470	KF926471	-	0.8	3.3	5.8	3.0	8.0	1.2	4.0	2.4
MYC79	Hosszúpályi	2011	Lung	KJ438257	KJ438194	KJ438215	KJ438236	-	0.8	3.3	5.8	3.0	8.0	1.2	4.0	2.4
MYC80	Ebes	2011	Lymphnode	KF926472	KF926473	KF926474	KF926475	2.2	0.8	3.3	-	-	25.0	1.2	4.0	2.4

Herd of origin, date, sample type, GenBank Accession numbers of locus sequences and number of TRs of the thirty-one *M. bovis* strains analyzed in this study.

The MLST based on four housekeeping genes (*fusA, gyrB, lepA, rpoB*) was performed using the amplification primers and PCR conditions described by Manso-Silván et al. [10] in 25 μl total volume containing 10 to 100 ng of target DNA diluted in nuclease-free water, 5 μl of 5× Green GoTaq Flexi Buffer (Promega, Inc., Madison, WI), 2.5 μl of MgCl_2_ (25 mM; Promega), 0.75 μl of deoxynucleoside triphosphates (10 mM; Qiagen Inc.), 1 μl of each primer (10 pmol/μl), and 0.25 μl of GoTaq Flexi DNA polymerase (5 U/μl; Promega, Inc.). The PCR were performed in a Biometra – T Personal thermal cycler (Biometra Inc., Göttingen, Germany). PCR products were isolated from agarose gel (QIAquick gel extraction kit; Qiagen Inc.), and direct cycle sequencing was performed with the primers used for amplification on an ABI Prism 3100 automated DNA sequencer (Applied Biosystems, Foster City, CA). The reading errors of the chromatograms were corrected with the SeqMan program (Lasergene package, DNASTAR Inc., Madison, WI). Sequences were trimmed, concatenated and aligned with all published sequences using the BioEdit 7.2.2 software [16].

An MLVA based on nine tandem repeats (TR)s was performed with the amplification primers and PCR conditions described by Pinho et al. [9]. All PCRs were performed in 25 μl total volume as described above. After amplification, 2–5 μl of each reaction mixture was subjected to electrophoresis (8 V/cm) in 2% standard agarose gel (SeaKem LE Agarose, Lonza Inc., Rockland, ME), and the amplified DNA products were visualized with GR Safe nucleic acid gel stain (Lab Supply Malla InnoVita Inc., Gaithersburg, MD). Depending on the length of the tandem repeat units a 100-bp or a 20-bp DNA ladder (GeneRuler 100 bp Plus or O'RangeRuler 20 bp, Thermo Fisher Scientific Inc.) was used as molecular weight marker. Electrophoresis was performed until the yellow dye had run for at least 20 cm. Stained gels were visualized by UV light, photographically documented (Kodak Inc., Rochester, NY) and band sizes were estimated with the help of the Kodak MI SE software package (Kodak Inc.).

Phylogenetic analysis of the concatenated sequences containing the four housekeeping genes was conducted with the neighbour-joining method using pairwise distances and 1000 bootstraps in the MEGA 5.05 software [17]. The average evolutionary divergence of the concatenated sequences was also estimated with MEGA 5.05 [17] both within and between *M. bovis* clades. Analyses were conducted using the Maximum Composite Likelihood model with standard error estimated through 1000 bootstrap replicates. The rate variation among sites was modelled using gamma distribution (shape parameter = 1) including all codon positions. A recombination analysis was performed on the concatenated sequence alignment using the RDP 4 software [18]. The default selection of detection methods (RDP, GeneConv, and MaxChi) and general settings were used to perform the analyses. In MLVA, the band size estimates were converted to numbers of repeat units [9]. The clustering analysis was performed with a neighbour-joining method based on pairwise distances in the MEGA5.05 software [17]. The discriminatory power of the different typing schemes was calculated using Simpson’s index of diversity with 95% confidence intervals (CI) [19]. The quantitative level of congruence and respective confidence intervals between the two typing methods was calculated based on the data of the thirty-one isolates and PG45 analyzed with both methods using the adjusted Rand and Wallace coefficients [20]. An online tool was used to perform these calculations [21].

## Results

**Figure 1:**
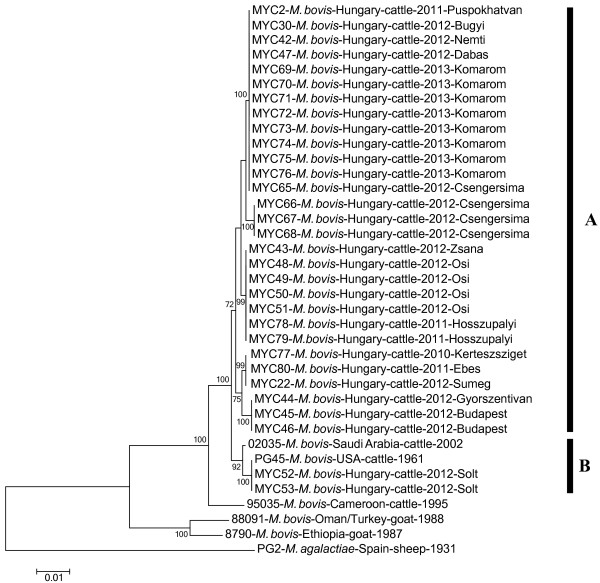
**Genetic relationships between *M. bovis *strains based on multi locus sequence typing.** Neighbour-joining phylogenetic tree showing relationships between the concatenated partial sequences of four housekeeping genes obtained from *M. bovis* strains characterized in this study (Hungary) and deposited in GenBank. Panels **A** and **B** indicate the major clades. Bootstrap values of neighbour-joining (1000 replicates) of > 70 are shown. The scale bar represents the average number of substitutions per site.

Hungarian *M. bovis* isolates clustered into two clades by MLST (Figure 1). Two strains (MYC52, 53) had the same sequence type (ST) as the reference strain PG45 (NCTC 10131) and were closely related to a strain from Saudi Arabia, while the other Hungarian isolates formed a novel clade with five subclades. Isolates originating from the same herds had the same STs and were assigned to the same subclades by MLST except for one strain (MYC 65). The within-group means of genetic distances between the concatenated sequences were 0.001 (±0.001 standard error, SE) in the Hungarian clade (Clade A) and 0.001 (±0.000 SE) in the PG45 clade (Clade B). The mean values of between clade genetic distances for the concatenated sequences ranged from 0.003 to 0.012 (Table 2). The recombination analyses on the alignment of the concatenated MLST loci did not reveal any recombination events.

**Table 2:** **Estimates of average evolutionary divergence of concatenated sequence pairs between and within *M. bovis *** **clades**

	**Evolutionary divergence**					
	**Clade A**	**Clade B**	**M.b.-Cameroon**	**M.b.-Oman/Turkey**	**M.b.-Ethiopia**	**Distance (SE)**
Clade A						0.001 (0.001)
Clade B	0.003					0.001 (0.000)
M.b.-Cameroon	0.006	0.005				n/c
M.b.-Oman/Turkey	0.012	0.012	0.010			n/c
M.b.-Ethiopia	0.012	0.012	0.010	0.003		n/c
M. agalactiae	0.030	0.030	0.029	0.029	0.027	n/c

The numbers of base substitutions per site are shown as calculated from the average substitutions of all sequence pairs between (matrix) and within (column) groups. Standard error estimates are shown in parentheses. n/c: not calculated.

**Figure 2:**
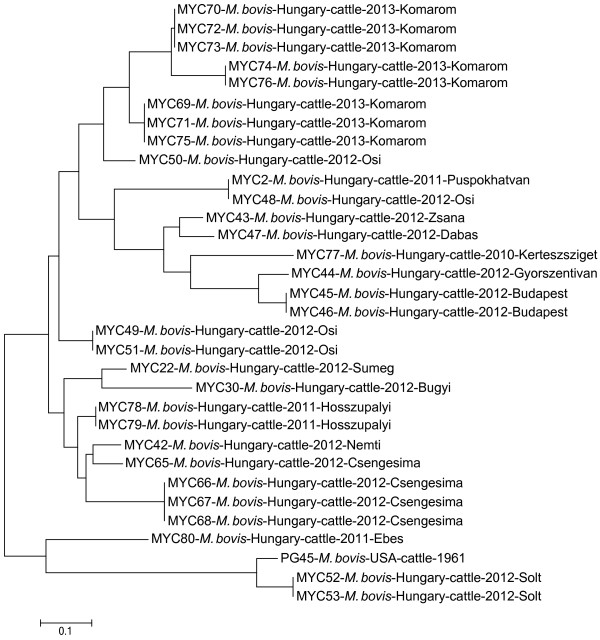
**Genetic relationships between *M. bovis *strains based on multiple-locus variable-number tandem repeat analysis.** Genetic relationships between thirty-one Hungarian isolates and the reference strain PG45 based on multiple-locus variable-number tandem repeat analysis. Dendrogram was constructed with the neighbour-joining method based on pairwise distances. The scale bar represents the average number of substitutions per site.

The Hungarian *M. bovis* isolates and the reference strain PG45 formed two major clades with several subclades and branches by MLVA (Figure 2). One main group comprised three strains (MYC 52, 53 and 80) and the reference strain PG45 while the other main branch contained the rest of the strains. Isolates originating from the same herd were generally clustered together or close to each other (MYC 45–46, MYC 52–53, MYC 65–68, MYC 69–76, MYC 78–79), but within herd divergence was also detected (MYC 48–51).

Six STs were discriminated by MLST and twenty different MLVA profiles were identified among the analyzed strains and PG45. The Simpson's index of diversity was 0.776 (CI: 0.678-0.874) in MLST and 0.970 (CI: 0.952-0.987) in MLVA. The value of the Adjusted Rand coefficient was 0.178, the Adjusted Wallance coefficient MLVA → MLST was 0.099 (CI: 0.004-0.194) and the Adjusted Wallance coefficient MLST → MLVA was 0.914 (CI: 0.828-1.000). These values indicate poor concordance between the results of the two typing systems.

Correlation was not found between the genotype and source (lung, nasal swab or lymph node) of the *M. bovis* isolates.

MLST alignment, MLVA data and phylogenetic trees were deposited in Dryad Digital Repository ([22], doi:10.5061/dryad.f4ks8).

## Discussion

*M. bovis* can cause great economic losses in the cattle industry through reduced production, increased costs of treatment and death. The disease is primarily transmitted by direct contact and it is spread through the movement of infected animals; therefore efficient genotyping tools are essential for its monitoring, control and epidemiological investigation. MLST and MLVA of *M. bovis* had been recently developed and they proved to be useful in previous studies [9,10,13,14]. The two methods differ considerably in their applicability. MLST is dedicated to the discovery of intermediate-term evolutionary events while MLVA is a suitable method to perform short-scale epidemiology studies. In the present work we tested these methods on a *M. bovis* strain collection isolated over the last couple of years in Hungary.

MLST results provided an intermediate level genetic resolution among the studied *M. bovis* strains. Isolates originating from the same farms had identical STs except for one case. Strains with distinct origins (e.g. when *M. bovis* infection might have been introduced to the herd through more than one animal of different origin) could explain the case of this exception (Csengersima, MYC 65–68). It is remarkable that the majority of the Hungarian isolates had different STs than the previously examined French, Belgian and German isolates which possessed the same sequence as PG45 [10]. However, it has to be noted, that only one isolate per country was examined in that particular study. Furthermore, unexpectedly high genetic variation was detected among the Hungarian strains by MLST. Similar analyses would be required on strains originating from other countries to explore whether the MLST system really possesses such discriminatory power or whether it is the Hungarian *M. bovis* population that is genetically so diverse. Potentially the high genetic diversity of the Hungarian *M. bovis* population may be partially due to the intensive national and international cattle trade.

It was demonstrated that MLVA has high discriminatory power and is able to distinguish closely related strains. This was best demonstrated during the typing of isolates from the herd from the town of Komárom (MYC 69–76). These strains formed one group with three subgroups. Spergser et al. [13] modified the MLVA typing scheme described by Pinho et al. [9]. With their modification Spergser et al. [13] increased the discriminatory power (Simpson's index of diversity) of the original MLVA system from 0.952 to 0.981 based on the analysis of their samples. In the present study we analyzed our samples with the original Pinho et al. [9] system resulting a 0.970 Simpson's index of diversity. This value provides further proof for the high genetic diversity of the Hungarian *M. bovis* population. This finding is consistent with previous data published by Amram et al. [14] who discovered eight different VNTR patterns among the eleven *M. bovis* strains isolated from calves imported to Israel from Hungary. Unfortunately we were unable to compare our isolates with the strains analysed by MLVA in the previous studies [9,13,14], as the VNTR patterns of the individual isolates from these studies were not published. Establishing an online database of *M. bovis* VNTR patterns or providing supplementary material with publications would allow the comparison of isolates originating from different parts of the world, necessary for the monitoring of the disease and for performing epidemiological investigations. Furthermore, the availability of a large dataset should also improve the methods for data analysis. Although we think that MLST is a more appropriate method than MLVA for the genetic comparison of weakly related isolates (strains with distant geographic origin, e.g. on a country or continental level), it may also be possible to use the later for this purpose with certain modifications. As MLVA is primarily a fine scale typing tool (e.g. useful in following local epidemics), the different TRs are recommended to be weighted when the method is applied on weakly related isolates in order to avoid homoplasy [23]. However, in order to establish an efficient TR weighting system, it is essential to have access to VNTR profile data of many isolates from different countries.

## Conclusions

**Figure 3:**
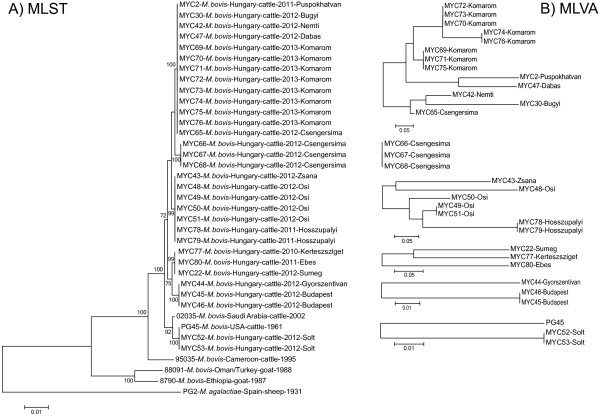
**Genetic relationships between *M. bovis *strains based on the combined use of multi locus sequence typing (MLST, A) and multiple-locus variable-number tandem-repeat analysis (MLVA, B).** Dendrograms were constructed with neighbour-joining methods. Scale bars represent the average number of substitutions per site.

This study has compared and evaluated two different molecular epidemiological typing techniques for *M. bovis* characterisation. MLST is a robust and reproducible intermediate scale typing method which proved to be discriminatory among the Hungarian *M. bovis* isolates. MLVA is an appropriate fine-scale typing tool for *M. bovis* strains, as it even allowed the within farm differentiation of certain strains*.* Based on these results the combined use of the two typing methods is recommended (Figure 3). The isolates have first to be typed by MLST and then the subset of strains sharing the same STs has to be further differentiated by MLVA. In this way information will be gained about both the longer-term evolutionary and the short-term epidemiological relationships of the analysed isolates.

## Abbreviations

MLST: Multi locus sequence typing; MLVA: Multiple-locus variable-number tandem repeat analysis; ST: Sequence type; VNTR: Variable-number tandem repeats.

## Competing interests

The authors declare that they have no competing interests.

## Authors’ contributions

KMS and ZK performed MLST and MLVA, analysed the data and wrote the manuscript. LF performed MLST and MLVA. SJ, NS, IT and LM collected the samples and isolated the strains. KE analysed the data and wrote the manuscript. MG designed the study, analysed the data and wrote the manuscript. All authors read and approved the final manuscript.
